# Supplementary material for: DHODH Inhibition Exerts Synergistic Therapeutic Effect with Cisplatin to Induce Ferroptosis in Cervical Cancer through Regulating mTOR Pathway
Source: Cancers (Basel). 2023 Jan 16;15(2):546. doi: 10.3390/cancers15020546 (PMC9856746; doi:10.3390/cancers15020546)
Supplement: Supplementary file 1 [file cancers-15-00546-s001.zip › Figure S1.pdf]

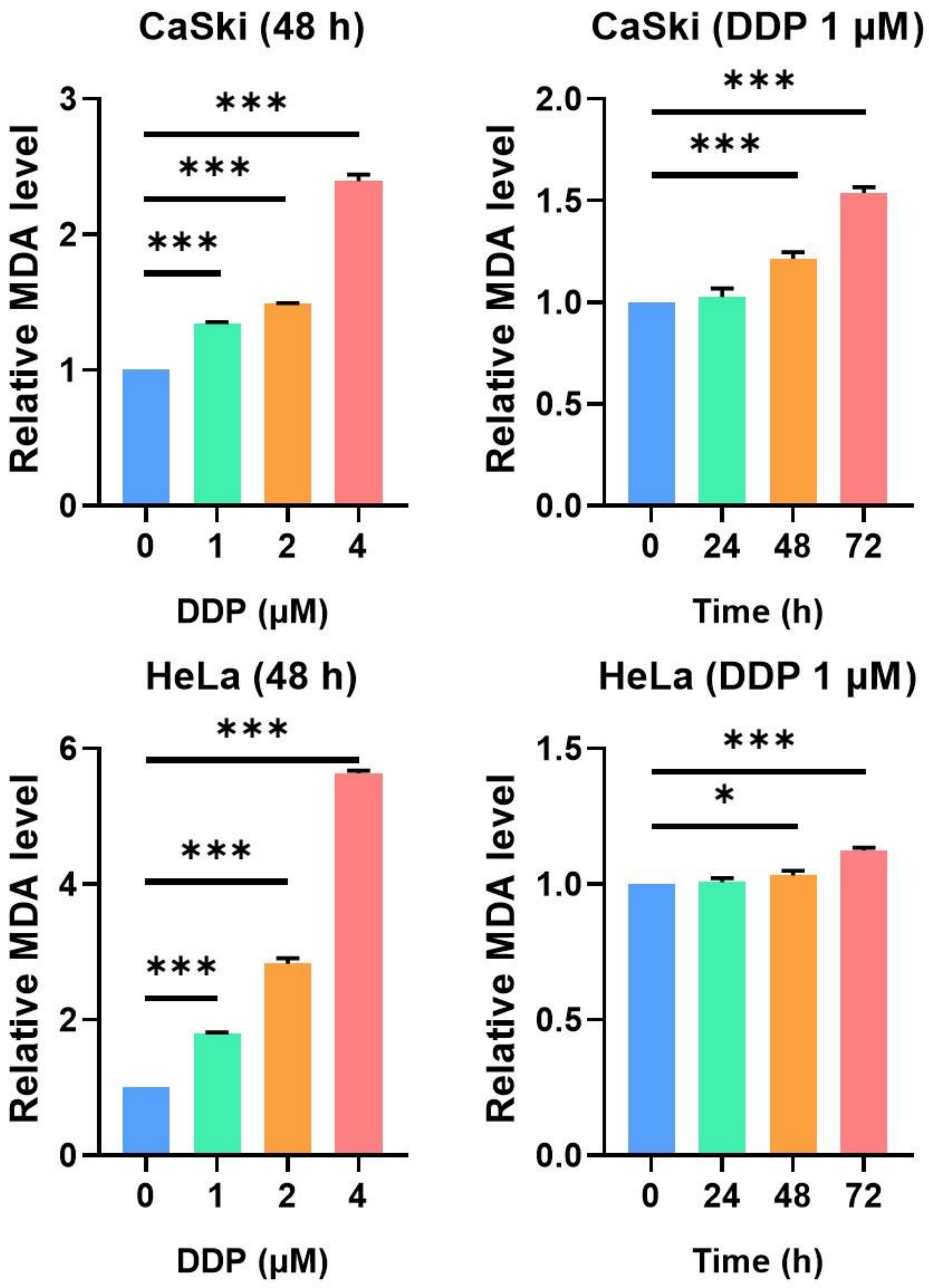

Figure S1. Cisplatin induces ferroptosis in a dose- and time-dependent way in cervical cancer cells. * $p < 0.05$, *** $p < 0.001$ compared to the control group.
