# Supplementary material for: DHODH Inhibition Exerts Synergistic Therapeutic Effect with Cisplatin to Induce Ferroptosis in Cervical Cancer through Regulating mTOR Pathway
Source: Cancers (Basel). 2023 Jan 16;15(2):546. doi: 10.3390/cancers15020546 (PMC9856746; doi:10.3390/cancers15020546)
Supplement: Supplementary file 1 [file cancers-15-00546-s001.zip › Figure S2.pdf]

Figure 1B, CaSki cells (DHODH; GAPDH)

1.

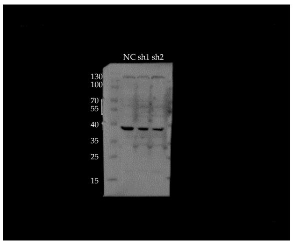

DHODH;

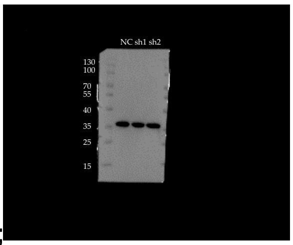

GAPDH;

2.

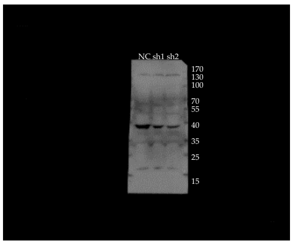

DHODH;

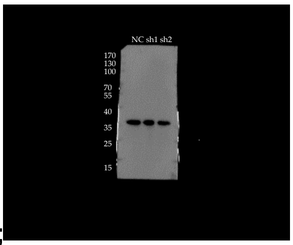

GAPDH

3.

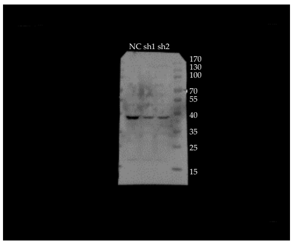

DHODH;

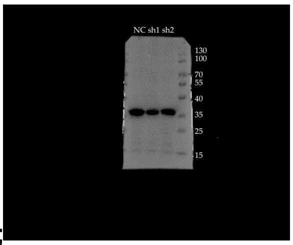

GAPDH

Figure 5A, CaSki cells (DHODH; mTOR; p-mTOR; GAPDH; Vinculin)

1.

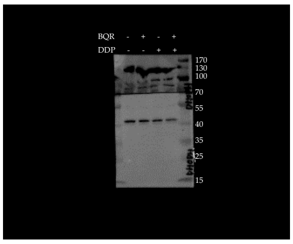
DHODH;

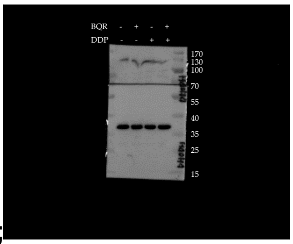
GAPDH;

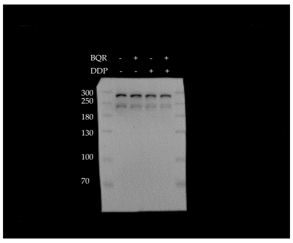
mTOR;

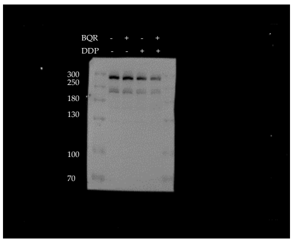
p-mTOR;

2.

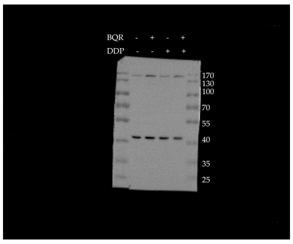
DHODH;

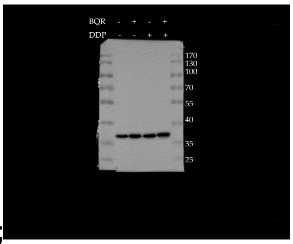
GAPDH;

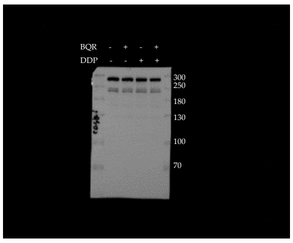
mTOR;

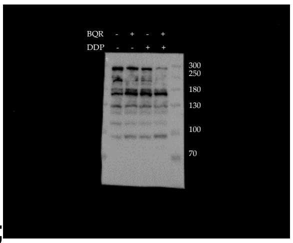
p-mTOR;

3.

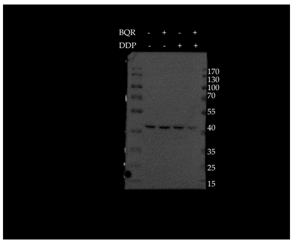
DHODH;

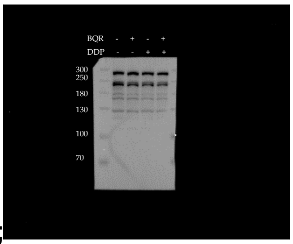
mTOR;

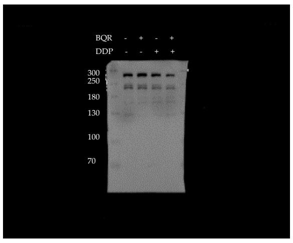
p-mTOR;

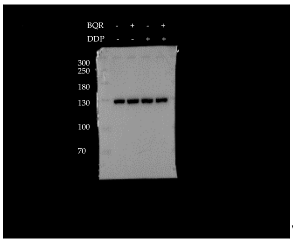
vinculin

Figure 1B, HeLa cells (DHODH; GAPDH)

1.

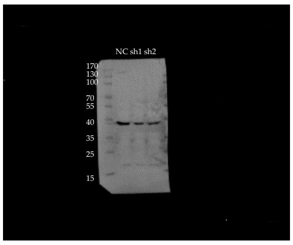

DHODH;
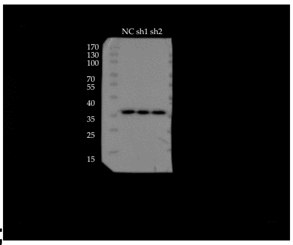

GAPDH

2.

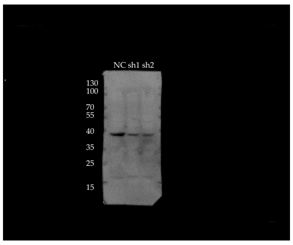

DHODH;
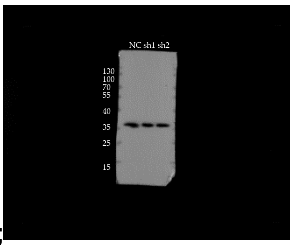

GAPDH

3.

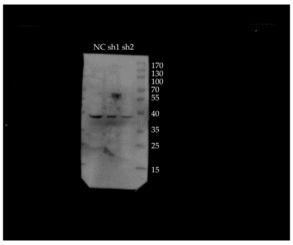

DHODH;
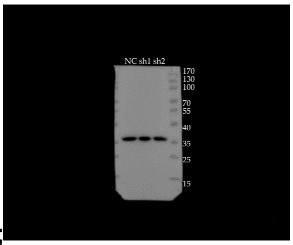

GAPDH

Figure 5A, HeLa cells (DHODH; mTOR; p-mTOR; GAPDH; Vinculin)

1.

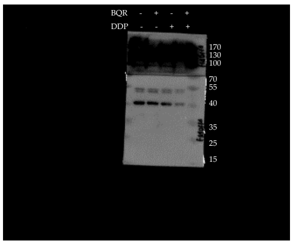
DHODH;
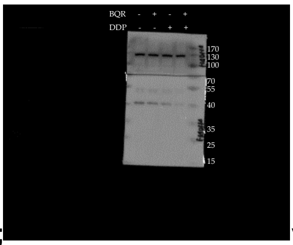
vinculin;

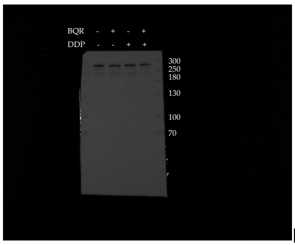
mTOR;
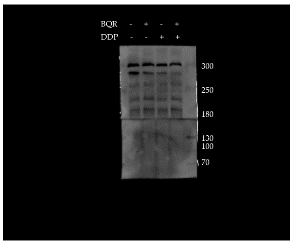
p-mTOR;

2.

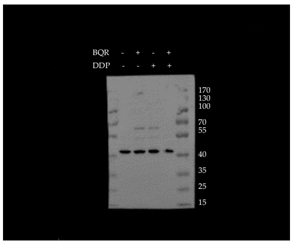
DHODH;
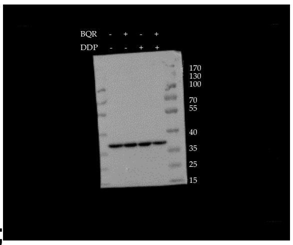
GAPDH;

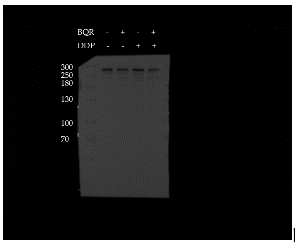
mTOR;
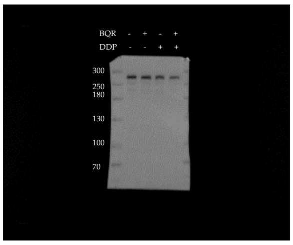
p-mTOR;

3.

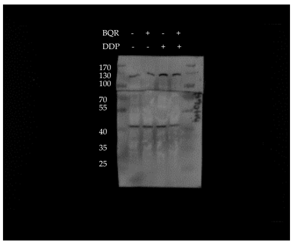
DHODH;
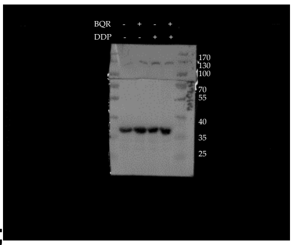
GAPDH;

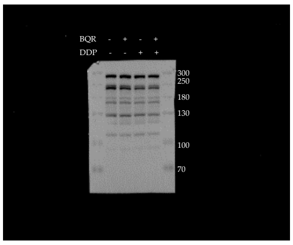
mTOR;
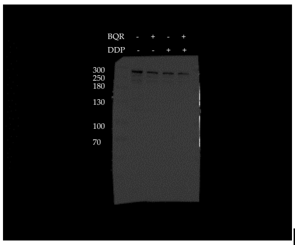
p-mTOR;

Figure S2. Original membranes of immunoblots shown in Figures 1B and 5A.
